# Predation Risk Effects of Lady Beetle *Menochilus sexmaculatus* (Fabricius) on the Melon Aphid, *Aphis gossypii* Glover

**DOI:** 10.3390/insects15010013

**Published:** 2023-12-27

**Authors:** Xingming Lin, Xiangxin Cui, Jihong Tang, Jiawei Zhu, Jinhua Li

**Affiliations:** 1 Key Laboratory of Green Prevention and Control of Tropical Plant Diseases and Pests, Ministry of Education, School of Tropical Agriculture and Forestry, Hainan University, Haikou 570228, China; lxmsunny@foxmail.com (X.L.); cui18703668762@163.com (X.C.); zhuhh86@163.com (J.Z.); 2 Key Laboratory of Integrated Pest Management on Tropical Crops of Ministry of Agriculture and Rural Affairs, Environment and Plant Protection Institute, Chinese Academy of Tropical Agricultural Sciences, Haikou 571101, China; jihong_23@163.com

**Keywords:** *Menochilus sexmaculatus*, *Aphis gossypii*, predation risk, host preference, defense enzyme activities, fitness

## Abstract

**Simple Summary:**

Biological control is an important method for pest management. Natural enemies not only prey on pests directly but also release predation risk cues to pests, causing physiological and behavioral changes in them, reducing their fitness, and ultimately leading to a decrease in their population. *Aphis gossypii* is an important insect pest of melon and many other crops worldwide. This study examined the predation risk effects of *Menochilus sexmaculatus*, which is abundant in agricultural areas of southern China, on *A. gossypii*. The results showed that the presence of *M. sexmaculatus* significantly altered the host preference of the aphids. The longevity of the aphids was shortened, and the reproductive period and offspring production were decreased significantly after exposure to *M. sexmaculatus* for 24 h. The enzyme activities of SOD and CAT were increased, and the content of MDA in aphids also showed an increase under the predation risk of *M. sexmaculatus*. Through a combined GC-MS and behavioral response experiment, the results showed that alkane compounds may mediate the predation risk. The results will offer an alternative strategy for utilizing these alkanes as repellents to manage aphids.

**Abstract:**

Predation risk posed by natural enemies can alter pest performance. In our previous study, we found *Menochilus sexmaculatus* provides risk cues to melon aphids, resulting in increased numbers of winged aphids. However, the effects of predation risk on multiple traits including behavior, physiology, growth rate, and reproductive capacity of pests are not clear. This study examined the effects of predation risk on host preference, the activities of two important defense enzymes (CAT and SOD), longevity, and offspring production. The Y-tube trial results showed that the risk of *M. sexmaculatus* significantly altered the host preference of the aphids, leading to avoidance behavior. When exposed to *M. sexmaculatus* for a long period (24 h), the reproductive period and offspring production were significantly decreased, and adult longevity was significantly shortened. The defense enzyme activities of SOD and CAT, as well as the MDA content (which is considered a marker of oxidative stress and cellular damage) in the aphids, significantly increased under *M. sexmaculatus* risk. The compounds of *M. sexmaculatus* extracted with n-hexane and volatile compounds collected with HS-SPME were analyzed by GC-MS, and when combined with the behavior response experiment, the results showed that the alkane compounds *n*-henicosane, *n*-docosane, *n*-tricosane, *n*-pentacosane, and *n*-hentriacontane may contribute to the impact of predation risk. The results will be helpful in the comprehensive evaluation of the ability of lady beetles to affect the aphid population, and provide new ideas for using these compounds in aphid control.

## 1. Introduction

Biological control plays a crucial role in reducing the need for conventional chemical insecticide applications, which may lead to various well-known issues like the development of insecticide resistance and the creation of ecological hazards [1,2]. Traditional biological control primarily relies on the direct predation of pests by natural enemies to decrease prey demography. In addition to this direct predation, predation risk effects can also be utilized to regulate pest populations, making them a crucial aspect of integrated biological control advancement. The effects of predation risk are the changes in prey behavior, physiology, development, and morphological traits when they respond to the perceived threat of predation, in another words, it means anti-predation responses [3]. Predation risk does not kill the prey, but it may lead to negative consequences including increased stress levels and alterations in prey fitness or migration patterns, reflected in changes in prey feeding habits, spawning behaviors, colonization tendencies, dispersal patterns, and host preferences, and these indirect effects may reduce the suitability of pests [4].

The ability of prey to perceive the risk of a given predator species is a prerequisite for predation risk effects. Prey can detect predators using various sensory cues, including visual, tactile, vibrational, and chemical cues. Among these, volatile chemical cues have been reported to be particularly important and have been implicated in many predator-prey predation risk effects [5]. For example, the potato beetle *Leptinotarsa deceminineata* Say (Coleoptera: Chrysomelidae) reduced its intake by 29% under the predation risk from the volatiles of *Podisus maculiventris* Say (Hemiptera: Pentatomidae) [6]. The chemical cues of *Paederus fuscipes* Curtis (Coleoptera: Staphylinidae) increased the feeding behavior, growth, and development rate of *Laodelphax striatellus* Fallen (Homoptera: Delphacidae) nymphs, shortened their adult lifespan, and decreased their fecundity [7]. When *Culiseta longiareolata* Macquart (Diptera: Culicidae) perceived the volatile of natural enemy *Notonecta maculate* Fabricius (Hemiptera: Nepomorpha), evasive behavior was induced, and the spawning frequency was decreased [8,9].

Lady beetles, which are one group of important natural enemies, feed on various pests, including aphids, thrips, mites, and scale insects. Besides direct predation, predation risk effects can also be induced when prey is exposed to lady beetles. The predation risk of *Harmonia axyridis* Pallas (Coleoptera: Coccinellidae) alters the host preference of the aphid *Myzus persicae* Sulz. (Hemiptera: Aphididae), reduces the offspring, and increases the number of winged aphids in the offspring [10]. The chemical from the footprint of *Coccinella septempunctata* Linnaeus (Coleoptera: Coccinellidae) has been shown to reduce the number of aphids produced by *Rhopalosiphum padi* Linnaeus (Hemiptera: Aphididae) [11]. *Menochilus sexmaculatus* Fabricius (Coleoptera: Coccinellidae), which is abundant in agricultural areas of southern China, has demonstrated higher predation rates on aphids compared to many other coccinellids such as *Coccinella transversalis* Fabricius (Coleoptera: Coccinellidae) and *Propylea dissecta* Mulsant (Coleoptera: Coccinellidae). This indicates its effectiveness as a biological control agent for aphids [12]. *M. sexmaculatus* also showed predation risk, producing predation risk effects on their prey. The chemical compounds in the footprints left by this lady beetle have been shown to alter the feeding rate, conversion efficiency, and growth rate of the leaf beetle *Zygogramma bicolorata* Pallister (Coleoptera: Chrysomelidae) [13].

Cucumber *Cucumis sativus* L. belongs to the family of Cucurbitaceae [14,15]. In 2021, The world cultivation area of the cucumber was 2.172 million hectares and the annual cucumber output was 93.53 million metric tons. The yield and quality of cucumbers are often influenced by different biological factors. *Aphis gossypii* Glover (Homoptera: Aphididae), one of the most serious pests in cucumber that often causes severe damage to cucumbers [16,17,18], has been regarded as a cosmopolitan, highly polyphagous species, widely distributed in tropical, subtropical, and temperate regions [19]. In a previous study, we found that *M. sexmaculatus* also provides risk clues to melon aphid *A. gossypii,* leading to an increase in the number of winged aphids (unpublished data). Consideration of how multiple traits might change in prey organisms is crucial to understanding the impact of predation risk on overall fitness [20,21]. To explore the multiple traits that might change in *A. gossypii,* in the present study, the effects of predation risk of *M. sexmaculatus* on aphid behavior, physiology, growth, and reproduction were determined, and with GC-MS, the active compounds were identified. The results reveal the mechanism of predation risk of *M. sexmaculatus* on the aphid *A. gossypii*. This research will contribute to a more comprehensive evaluation of the population regulation ability of lady beetles on aphids and provide new ideas for the environmentally friendly prevention and control of aphids, especially those that are resistant.

## 2. Materials and Methods

### 2.1. Aphids for Laboratory Experiments

The melon aphid *A. gossypii* was collected from the leaves of cucumber at the Agricultural Science Base of Hainan University (20°05′N, 110°32′E) Haikou, China, during December 2018. Collected individuals were used to establish a colony in controlled conditions. *A. gossypii* were reared on cucumber *C. sativus* plants in a rearing room (26 ± 1 °C, 16:8 h L:D, 70% ± 5% RH). Each cucumber plant was grown in a plastic pot (15 cm high, 10 cm diam.) with standard potting soil.

### 2.2. Menochilus sexmaculatus for Laboratory Experiments

Adult *M. sexmaculatus* was collected from a cornfield of Hainan University (20°05′N,110°32′E) in December 2018. Wild-caught *M. sexmaculatus* were subsequently released onto the cowpea Vigna unguiculata seedlings that had sufficient aphid populations for sustenance. The cowpea plants, along with the aphids, were placed inside a gauze cage (40 × 40 × 40 cm). Egg laying by the female was checked every 24 h, and the leaves with eggs were collected and kept in a petri dish for hatching. After hatching, *M. sexmaculatus* larvae were individually fed with *A. craccivora* in petri dishes (d = 9 cm), and kept at 26 ± 1 °C, 16:8 (L:D) condition. Old and dead aphids were removed from the petri dish daily. When *M. sexmaculatus* larvae entered the pupal stage, the supply of aphids was stopped. After approximately 6–10 days, the emerged adults were transferred into the cages (40 × 40 × 40 cm), and they were provided *A. craccivora* for feeding continuously.

### 2.3. Behavioral Responses of Aphis gossypii to Menochilus sexmaculatus Risk

To explore the effects of *M. sexmaculatus* risk on *A. gossypii* feeding choice preference, behavioral experiments were performed using a glass Y-tube olfactometer (Longteng Medical Glass Company, Harbin, China) (with a 0.5 cm internal diameter, 6 cm trunk length and 6 cm branch length). A Y-shaped copper wire is put in the center of the Y tube to facilitate the movement of the aphids. Odor sources were placed in a 125 mL scrubbing bottle and connected to 0.1 L/min, charcoal-filtered, and humidified airflow. Two separate odor source arenas were set up in tandem, one for control and one for treatment. Control and treatment arenas were then connected via silicone tubing with each odor source supplementing airflow to an individual arm at the end of a y-shaped olfactometer (y-tube). In this way, each arm of the “Y” consisted of a distinct odor source that flowed down towards the base of the “Y” where insects were released and left to make a choice. For each experimental replicate, a single adult aphid was selected randomly and placed at the open end of the olfactometer with a fine-tipped paintbrush. The movement of aphids towards either the treatment or control arm was observed for a maximum 15 min [22]. A choice was recorded when *A. gossypii* moved at least halfway up one of the branched arms of the olfactometer, which means the choices of aphids in each arm exceeded 3 cm were recorded. The treatment and control tubes were switched from the right to the left arm of olfactometer prior to each two trials to reduce positional bias. All trials were conducted between 9:00 and 12:00 h [20]. Temperature of 26 ± 1 °C and RH of 60 ± 10% were maintained as experimental conditions. The treatment odor source including three groups: third-instar larvae, female, and male. To allow for lady beetle acclimatization and the accumulation of volatile cues, the lady beetle was placed in the scrubbing bottle of odor sources for 12 h prior to the aphid choice experiment. Seven replications were carried out for each group, with each replication consisting of 15 apterous aphids, in total 105 aphids used for each treatment. After each replicate, the y-shaped olfactometer was washed with acetone and left to dry, ensuring that aphids were not influenced by the movement of their conspecifics in the glassware during previous replicates. Odor sources were changed after every replicate.

### 2.4. The Effect of Menochilus sexmaculatus Risk on Aphid Biology

The influence of the predation risk on *A. gossypii* exposed to *M. sexmaculatus* was carried out in a double-deck petri dishes system. This double-deck experimental arena consisted of a flat-bottomed dish at the top and bottom, and in the middle was a flat dish with a hole (d = 4 cm) in the center, which was covered with gauze mesh (fixed with hot melt glue). They were enclosed together with sealing film to form an enclosed cell. In the predation risk test, a leaf disc with aphids was placed in the bottom petri dish (d = 9 cm), and a moistened filter paper was provided to keep the leaf moist. For the risk treatment, a lady beetle was released into the top portion of the petri dish space where *M. sexmaculatus* could not consume the aphid as they were separated by the gauze mesh, which allowed olfactory cues to pass through to the aphids. For controls, predators were not released.

To assess the transgenerational effects of predation risk treatment on aphid biology under varying durations of predation risk exposure, 20 adults of *A*. *gossypii* were exposed to a single third-instar larvae of *M. sexmaculatus*. After being exposed to varying durations of *M. sexmaculatus* risk treatments (2, 6, and 24 h), adult aphids were allowed 24 h to produce nymphs. The newly born nymphs were individually transferred onto fresh cucumber leaves kept moist with 0.6% agar medium (one aphid per petri dish, d = 3.5 cm), and their survival, reproductive performance, and developmental period were recorded daily. Each treatment had three replications, with 20–25 apterous aphids in each replication. The cucumber leaves were replaced with fresh ones every 2 days until the aphids died. The petri dishes with aphids were kept under conditions of 26 ± 1 °C and RH of 60 ± 10%.

### 2.5. Oxidative Stress Level and Oxidative Damage of Aphids under Menochilus sexmaculatus Risk

To assess the response of oxidative stress in *A. gossypii* under predation risk, Superoxide dismutase (SOD), catalase (CAT) activities, and Malondialdehyde (MDA) were measured using a superoxide dismutase assay kit (BC0170, Solarbio, Beijing, China), a catalase assay kit (BC0200, Solarbio, Beijing, China), and a Malondialdehyde assay kit (BC0020, Solarbio, Beijing, China). Aphids were exposed to third-instar *M. sexmaculatus* for 24 h using the double-deck petri dish system (see results, 24 h caused significant changes in aphid biology). After exposure to the lady beetle, aphids were immediately collected and homogenized in 0.5 mL of the extract provided in the assay kits. The aphid homogenate was centrifuged at 8000 g for 10 min at 4 °C, and the supernatant was analyzed for the activity of SOD, CAT, and MDA to assess the oxidative stress levels in the aphids. Ten mg of aphids were collected for each replication, with three replicates for both the predation risk and control treatments. The absorbance values of the SOD, CAT, and MDA extracts were measured at 560 nm, 240 nm, and 532 nm, respectively.

### 2.6. Identification of Compounds of Menochilus sexmaculatus

Two methods, including Headspace solid phase microextraction (HS-SPME) and solvent extraction, were used to collect lady beetle compounds. For HS-SPME, *M. sexmaculatus* female, male, and third-instar larvae (20 individuals) were transferred to glass bottles (d = 3 cm, h = 9 cm). Then the protective rigid needle tube of the SPME extractor (57324-U, Supelco, Wilmington, NC, USA) was inserted into the bottle and fixed on the iron stand, the coated extractor fiber was extended and fixed, the analytes partition into the headspace, and the SPME fiber adsorbs them onto its surface. The extraction was carried out for 4 h. The volatiles adsorbed to the SPME fiber were then analyzed with GC-MS. For solvent extraction, the chemical of lady beetles was extracted with n-hexane (GC ≥ 99%). *M. sexmaculatus* female, male, and third-instar larvae (20 individuals) were transferred to glass bottles (d = 3 cm, h = 9 cm), and extracted with 2 mL n-hexane for 3 h at 25 °C. The extracts underwent filtration with a filter membrane to remove impurities, and then were analyzed with an Agilent 7890B GC (Agilent, Santa Clara, CA, USA) equipped with an HP-5ms capillary column (30 m × 0.25 mm i.d., 0.25 μm film thickness). For GC-MS, the injector was splitless at 250 °C. Helium at 1 mL/min was used as the carrier gas. The oven temperature was maintained at 50 °C for 1 min, then programmed at 8 °C/min to 120 °C, and held at this temperature for 13 min. The mass spectra were recorded in the electron impact mode at 70 eV (Source temperature: 230 °C; scanned mass range: 35 to 500 amu). The preliminary identifications were determined by comparing the mass spectrometry (MS) data with published spectra (NIST, 2002). To confirm these preliminary identifications, peak enhancement was performed on a gas chromatograph (GC) using authentic samples sourced from commercial suppliers.

### 2.7. Behavioral Responses of Aphis gossypii to Compounds of Menochilus sexmaculatus

The behavioral response of *A. gossypii* to alkane compounds, which has been reported related to predation risk, was studied using the glass Y-tube olfactometer trial, following the same method as described in Section 2.3. the Test compounds *n*-Henicosane (99% purity, TCI, Shanghai, China), *n*-docosane (99% purity, Yuanye, Shanghai, China), *n*-tricosane (99% purity, Yuanye, China) and *n*-pentacosane (97% purity, Aladdin, Shanghai, China) were purchased.

### 2.8. Statistics

We employed chi-square tests to analyze the impact of lady beetle risk on host preference of aphids by analyzing the number of *A. gossypii* entering the control versus treatment arm in the Y-tube olfactometer bioassays. The null hypothesis was equal entrance by aphids into both arms of the olfactometer. We used *t*-tests to compare the longevity of aphids, the reproduction period, and the number of offspring produced between lady beetle risk treatment and the control group. We also compared the defense enzyme activities of SOD and CAT, as well as the content of MDA, between the lady beetle risk and control groups using *t*-tests. We analyzed the number of *A. gossypii* entering the control arm versus the treatment arm using chi-square tests to verify the influence of alkane compounds on aphid behavior. SPSS Statistics 26 was used for statistical analysis.

## 3. Results

### 3.1. The Behavioral Response of Aphis gossypii to Menochilus sexmaculatus

**Figure 1:**
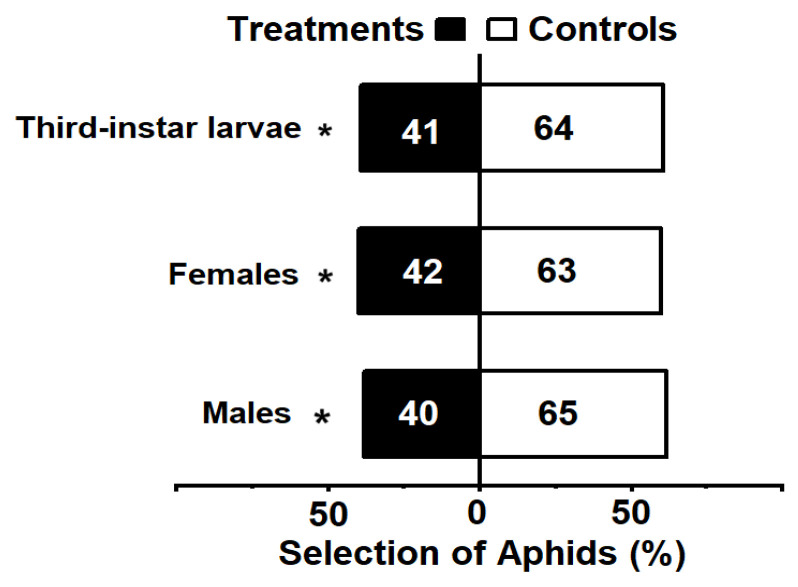
The choice of *Aphis gossypii* with a Y-tube olfactometer. Note: Bars represent the overall percentages of aphids choosing either of the odor sources; the number in the bar indicates the total number of aphids choosing the arm. The white bar represents control and the black bar represents lady beetle risk treatment. An asterisk indicates significant differences in the number of *A. gossypii* entering the control arm compared to the treatment arm in the Y-tube olfactometer bioassays, as determined by chi-square tests (*p* < 0.05).

The Y-tube trial demonstrated that the presence of *M. sexmaculatus* significantly altered the host preference of the aphids, leading to aversion behavior. When exposed to male *M. sexmaculatus*, 62% of the aphid individuals preferred the control option, while 38% opted for the odor source emitted by male lady beetle (χ^2^ = 5.952, df = 1, *p* = 0.015). In the larvae experiment, 61% of the aphid individuals preferred the control option, while 39% selected the odor source emitted by third-instar larvae (χ^2^ = 5.038, df = 1, *p* = 0.025). Furthermore, in comparison to the control group, 40% *A. gossypii* individuals preferred the odor source containing volatiles of female lady beetle (χ^2^ = 4.200, df = 1, *p* = 0.040) (Figure 1).

### 3.2. The Influence of Menochilus sexmaculatus Risk on Growth and Fecundity of Aphis gossypii

**Figure 2:**
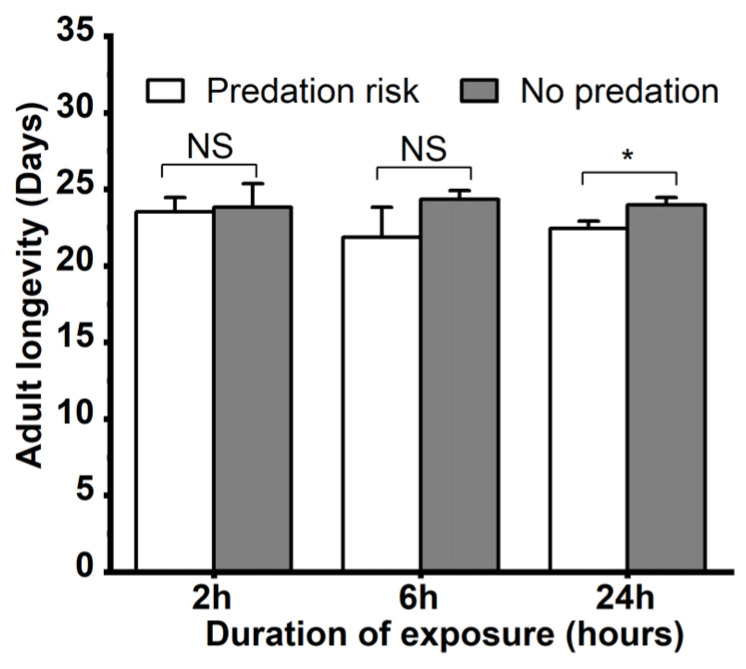
Adult longevity of *Aphis gossypii* under the predation risk of *Menochilus sexmaculatus.* Note: An asterisk placed between different treatments indicates significant differences in the same time period (*t*-test, *p* < 0.05), and NS represents no significant difference (*p* > 0.05).

The effects of *M. sexmaculatus* risk on the growth of *A. gossypii* were assessed. When exposed to *M. sexmaculatus* for different durations of time, the adult longevity and reproductive period of *A. gossypii* exhibited varying outcomes (Figure 2). The adult longevity of the control group was significantly longer than that of the aphids exposed to *M. sexmaculatus* for 24 h (t = 3.977, df = 4, *p* = 0.016). The mean longevity of adult progeny in the control group was 24.03 ± 0.49 days, which was significantly higher than the aphids exposed to *M. sexmaculatus* risk for 24 h (22.44 ± 0.49). However, there were no statistically significant differences between the control group and the treatment groups at other durations of predation risk exposure (2 h, 6 h; *p* > 0.05).

**Figure 3:**
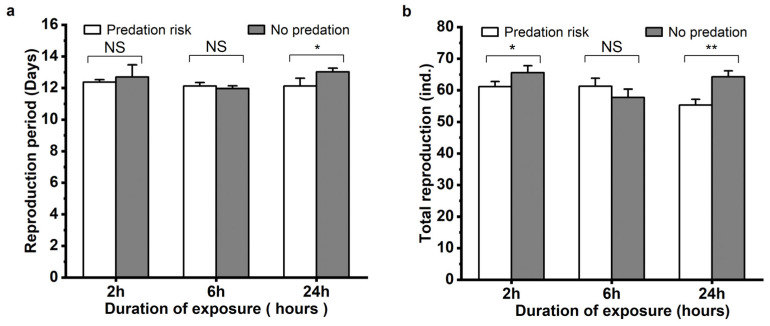
Reproductive period (**a**) and Total reproduction (**b**) of *Aphis gossypii* under the predation risk of *Menochilus sexmaculatus.* Note: Asterisks placed between different treatments indicate significant differences in the same time period (*t*-test, * *p* < 0.05; ** *p* < 0.01), and NS represents no significant difference (*p* > 0.05).

The effects of *M. sexmaculatus* risk on the reproduction of *A. gossypii* was also assessed, and the reproductive period and total aphid production of *A. gossypii* exhibited varying outcomes with different durations of predation risk (Figure 3). Compared to the control group, the total reproduction of the aphids under the predation risk of *M. sexmaculatus* significantly decreased by 6.77% after a 2 h treatment (t = 2.779, df = 4, *p* < 0.05). With a 24 h treatment of predation risk from *M. sexmaculatus*, the mean reproductive period also decreased significantly by 6.91% (t = 3.627, df = 4, *p* = 0.022), and the total reproduction of the aphids decreased by 13.96% (t = 5.892, df = 4, *p* = 0.004) under the predation risk treatment.

### 3.3. Oxidative Stress Level and Oxidative Damage of Aphis gossypii under Predation Risk

**Figure 4:**
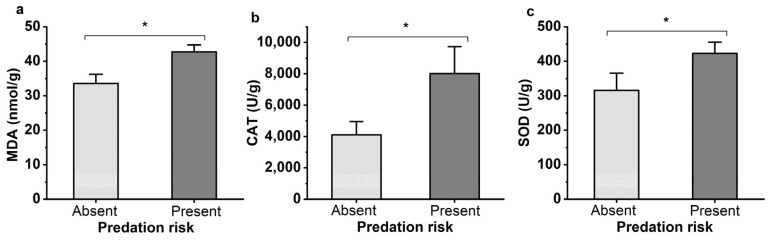
Oxidative damage to lipids (MDA levels, (**a**)), mean activity of defenses enzymes CAT (**b**) and SOD (**c**) of *Aphis gossypii* under the predation risk of *Menochilus sexmaculatus*. Note: An asterisk placed between different treatments indicates significant differences (*t*-test, *p* < 0.05).

The effects of *M. sexmaculatus* risk on the physiological status of *A. gossypii* were assessed by measuring the activities of two important defense enzymes (SOD and CAT), as well as the content of MDA, a biomarker of lipid peroxidation (Figure 4). After being exposed to the lady beetle *M. sexmaculatus* for 24 h, the MDA content of the aphids significantly increased by 27.30% compared to the control group (t = 4.880, df = 4, *p* = 0.008). The activity level of SOD was also significantly increased by 33.97% (t = 12.42, df = 4, *p* = 0.02). Under the stress of lady beetle, the enzyme level of CAT fluctuated greatly, with a 94.95% increase (t = 3.134, df = 4, *p* = 0.035).

### 3.4. GC-MS Analysis of the Compounds of Menochilus sexmaculatus

To identify the compounds of *M. sexmaculatus* responsible for *A. gossypii* changes, the n-hexane extracts and volatiles collected with HS-SPME were analyzed by GC-MS. The total compounds identified from extracted and volatile sources included various substances such as olefins, alkanes, amines, alcohols, esters, quinolizine, nitrogen-containing macrocycles, fatty acids, and others, resulting in a total of 42 compounds (Supplementary Tables S1 and S2). The alkanes primarily included *n*-henicosane, *n*-docosane, *n*-tricosane, *n*-pentacosane, and *n*-hentriacontane, which have been reported to induce predation risk in other species (Supplementary Figure S1, Supplementary Table S3).

### 3.5. Behavioral Responses of Aphis gossypii to Alkane Compounds

**Figure 5:**
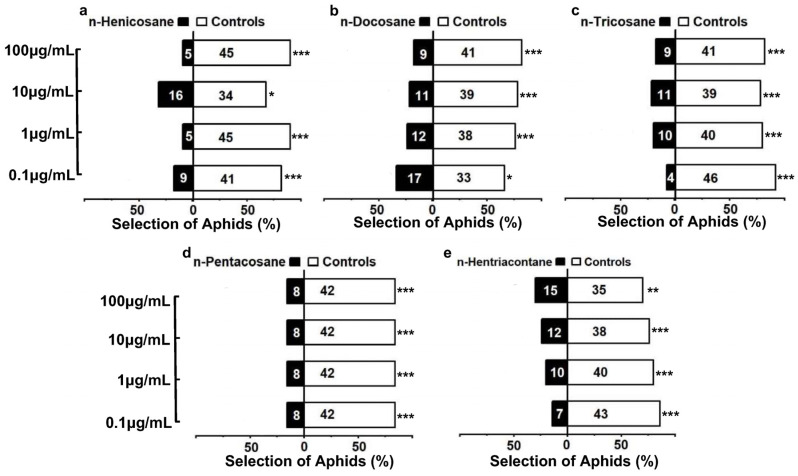
Behavioral responses of *Aphis gossypii* to various alkane compounds ((**a**). *n*-henicosane; (**b**). *n*-docosane; (**c**). *n*-tricosane; (**d**). *n*-pentacosane; (**e**). *n*-hentriacontane). Note: Bars represent the overall percentages of aphids choosing either of the odor sources; the number in the bar indicates the total number of aphids choosing the arm. The white bar represents control and the black bar represents alkane treatment. For the alkane treatments, from the top to the bottom represents the concentration from 100 μg/mL to 0.1 μg/mL. Asterisks indicate significant differences in the number of *A. gossypii* entering the control arm compared to the treatment arm in the Y-tube olfactometer bioassays (chi-square tests, * *p* < 0.05; ** *p* < 0.01; *** *p* < 0.0001).

To verify whether the alkanes contribute to the predation risk of *M. sexmaculatus,* the behavioral responses of *A. gossypii* to the compounds were conducted. The results showed that aphids exhibited avoidant responses to the alkanes (*n*-henicosane, *n*-docosane, *n*-tricosane, *n*-pentacosane and *n*-hentriacontane), and there were significant statistical differences in the number of selections between the odorant sources in the control group and the treatment group (Figure 5). The intensity of aphid reactions to different concentrations of *n*-henicosane chemical standards varies, ranging from 0.1 to 100 μg/mL. Among the four concentration gradients, the proportion of aphids choosing the alkane odor source was found to be 18% (χ^2^ = 20.48, df = 1, *p* < 0.0001), 10% (χ^2^ = 32.00, df = 1, *p* < 0.0001), 32% (χ^2^ = 6.48, df = 1, *p =* 0.011), and 10% (χ^2^ = 32.00, df = 1, *p* < 0.0001) respectively, indicating a relatively high avoidance effect of *n*-henicosane compared to the control. For *n*-docosane, the avoidance response increases with the increased concentration. In the highest concentration, 100 μg/mL, the strongest avoidance reaction of aphids was observed, with only 9 individuals (18%, χ^2^ = 20.48, df = 1, *p* < 0.0001) choosing the treated-arm. When the concentration of *n*-tricosane was 0.1 μg/mL, 8% aphids chose the treated odor source, while 92% aphids choose the control, indicating a significant avoidance response of the aphids to this alkane (χ^2^ = 35.28, df = 1, *p* < 0.0001). For other concentrations ranging from 1 μg/mL to 100 μg/mL, 20% (χ^2^ = 18.00, df = 1, *p* < 0.0001), 22% (χ^2^ = 15.68, df = 1, *p* < 0.0001) and 18% (χ^2^ = 20.48, df = 1, *p* < 0.0001) of the aphids chose *n*-tricosane treated odor. However, in the case of *n*-hentriacontane, the strongest avoidance reaction was observed at a concentration of 0.1 μg/mL. As the concentration increased from 0.1 μg/mL to 100 μg/mL, the proportion of aphids choosing the alkane odor source decreased to 14% (χ^2^ = 25.92, df = 1, *p* < 0.0001), 20% (χ^2^ = 18.00, df = 1, *p* < 0.0001), 24% (χ^2^ = 13.52, df = 1, *p* < 0.0001), and 30% (χ^2^ = 8.00, df = 1, *p* < 0.0001). Additionally, in the range of 0.1~100 μg/mL of *n*-pentacosane, there was no difference in the aphid selection response of different concentration gradients. A 16% aphid selection rate was observed for the treated odor source (χ^2^ = 23.12, df = 1, *p* < 0.0001), suggesting that their response was independent of concentration.

## 4. Discussion

Previous studies showed that if predation risk have a sufficient impact on pest fitness, a relatively small number of predators may be sufficient to keep pest populations below economic harm levels [23]. The effect of predation risk changes the growth, longevity, and reproductive ability of pests, and thus affect the population dynamics [24,25]. Many reports found that chemical cues can play a key role in the recognition and avoidance of predators and this has been well-documented in mammalian prey species [26,27]. As studies continue, more and more research reports that chemical cues also exist in arthropods. Chemical clues left by *C. septempunctata* reduced the colonization rate of *R. padi* by 40% [11]. *H. axyridis* crawling marks caused significant weight loss of *Acyrthosiphon pisum* Harris (Hemiptera: Aphididae) [28]. In the present study, we found that chemical cues can lead to predation risk effects and reduced the fitness of *A. gossypii*. When exposed to the chemical cues of *M. sexmaculatus*, *A. gossypii* exhibits altered behavior and physiology, which in turn impacts the growth and reproduction of the aphids.

Predation risk induced changes in various behaviors including host preferences, foraging, and oviposition behavior. For example, *L. deceminineata*, *Popillia japonica* Newman (Coleoptera: Scarabaeidae) and *Manduca sexta* Linnaeus (Lepidoptera: Sphingidae) reduced intake under the predation risk [6,29,30]. Larvae of the *Helicoverpa armigera* Hubner (Lepidoptera: Noctuidae) decrease their feeding time on host plants when predatory spiders are present [31]. Additionally, the predation risk posed by natural enemies has been shown to alter the oviposition behavior and host preferences on their prey [32,33]. In the present study, the volatiles of *M. sexmaculatus* significantly altered the host preference of *A. gossypii*, leading to an avoidance behavior. The avoidance effects were significant for lady beetle males, females, or third-instar larvae, with approximately 60% choosing the control (without the lady beetle). In another study, peach aphids *M. persicae* also showed a significant avoidant response towards lady beetle *H. axyridis* [10]. Aphids can recognize the volatiles emitted by lady beetle and will avoid areas where lady beetle are present. This behavior can benefit their offspring by providing a predator-free environment [34,35].

The avoidant response of aphid under predation risk may alter their physiology, including increased metabolic rates leading to an increase in ROS production. In order to maintain the balance between the generation and scavenging of ROS, energy consumption for the antioxidant enzyme synthesis system of aphids, and the activity levels of antioxidant enzymes SOD and CAT significantly increased [36]. These changes can be used to measure the changes in the physiology of aphids under predation risk [37,38]. In the present study, The MDA content of aphids under the predation risk of *M. sexmaculatus* also increased significantly. Although the synthesis of antioxidant enzymes was improved, it was not enough to eliminate the reactive oxygen species produced by oxidative stress. The aphids still suffered from oxidative damage. This is the same conclusion as has been previously reported [39,40,41].

Previous studies recognize that oxidative stress can be a mediator of trade-offs between life-history traits [42,43]. These physiological and behavioral changes indirectly reduce the suitability of pests, which may also have long-term irreversible effects on the life history of pests, such as the growth rate, life history, and the number and quality of offspring [44,45]. Stoks found that the predation risk stress of natural enemies may reduce the food digestion efficiency and metabolic rate of *Lestes sponsa* larvae, bringing down the development rate [46]. *Drosophila melanogaster* Meigen (Diptera: Drosophilidae) is stressed by mantis *Tenodera sinensis* Saussure (Mantodea: Mantidae) during the breeding period; the growth period of offspring was significantly shortened and the body weight decreased significantly [47]. In the present study, *A. gossypii* exposed to long-term (24 h) predation risk showed a significant reduction in reproductive performance, including reduced fecundity and a shortened reproductive period. These changes will eventually affect their population and play a positive role in biological control.

The changes of prey under predation risk may be related to the strength exerted by the predator. In the present study, long-term treatment (24 h) with lady beetle significantly led to changes in aphids, while short-term treatment (2 h and 6 h) had no significant effects on the aphids (except for total productivity at 2 h). Another study found that the effects of predation risk may depend on the density of ladybeetles. Low-density *H. axyridis* had no significant effect on pea aphid *A. pisum*, while medium-density and high-density lady beetles led to significant differences in fitness on pea aphids [48]. It was also found that different generations of aphids showed different responses under predation risk [49]. In natural ecosystems, natural enemies and pests have co-evolved over time, allowing pests to detect potential predators through various cues and adopt corresponding strategies. This process is influenced by several factors, including the species and density of the predator, the duration of predation-risk exposure, and the generation of pests. The process is complex, and its mechanism requires further exploration.

We identified the components of *M. sexmaculatus* using coupled GC-MS analysis, revealing the presence of *n*-henicosane, *n*-docosane, *n*-tricosane, *n*-pentacosane and *n*-hentriacontane in the lady beetle, which may contribute to the changes of aphids. Previous studies reported that alkanes including *n*-tricosane and *n*-pentacosane in lady beetle have biological activities that induce aphid avoidance behavior and relate to the effects of predation risk [50,51]. The effects of predation risk may not be related to the content of these compounds. In the present study, the behavior response of aphids treated with *n*-pentacosane, *n*-tricosane and *n*-henicosane did not exhibit a linear relationship with the concentration of these compounds. Indeed, for *n*-docosane, the proportion of aphids exhibiting avoidance response decreased with increasing concentration, while for n-hentriacontane, the proportion of aphids exhibiting avoidance response increased with increasing concentration. A study has found that an alkaloid called 2-methoxy-3-isopropylpyrazine, present in ladybeetles, exhibits high biological activity even at very low concentrations. It can be detected by human noses but cannot be detected by gas chromatography due to its low concentration [52]. Based on this, it can be inferred that no alkane compounds were extracted from the volatiles of female adults extracted by headspace solid-phase microextraction in this study, possibly due to the low release and volatilization of these compounds in females, while *n*-triacontane and *n*-pentacosane were separated from the volatiles of males, and *n*-pentacosane was separated from the third-instar larvae. From a holistic perspective, by combining the volatiles collected with HS-SPME and the extracts obtained with n-hexane, we found, in the present study, that these alkenes significantly contributed to the predation risk of *A. gossypii*, resulting in changes in aphid behavior and fitness.

It is worth mentioning that prey animals in the same predator-prey system may show different responses to male and female predators. For example, *P. maculiventris* reduced the intake of *L. decemlineata*, and this effect caused by males is stronger than that caused by females [6]. In the present study, the aphid response level for males, third-instar larvae and females of lady beetle was different slightly; the males provoke stronger aphid avoidance responses than the third-instar larvae, and the females are weaker. Previous studies showed that prey may detect male predators more easily and escape from them [53]. Females tend to release smaller bursts of volatiles to remain hidden and efficient, while males release more volatiles that travel longer distances [6,54]. However, some studies have shown that female predators may also release compounds that are more effective in inducing avoidance behavior in their prey, due to their need to mark their own active areas [55]. The specific volatile components emitted by different natural enemies can also lead to highly specific repellent reactions from their prey.

## 5. Conclusions

In summary, the presence of *M. sexmaculatus* significantly altered the host preference of the aphids, leading to avoidance behavior. The longevity of aphids was shortened, and their reproductive performance decreased significantly when exposed to *M. sexmaculatus* for a long term (24 h). Additionally, their physiology changed, including increased enzyme activities of SOD and CAT, as well as increased MDA content. An experiment combining GC-MS with behavioral responses suggested that alkane compounds (*n*-henicosane, *n*-docosane, *n*-tricosane, *n*-pentacosane, and *n*-hentriacontane) may contribute to the predation risk. The results will contribute to a more comprehensive evaluation of the ability of lady beetles to control aphid populations and provide new ideas for environmentally friendly methods of managing aphids in crops by using these alkanes as repellents.

## Data Availability

The data that support the findings of this study are available from the corresponding author upon reasonable request.

## Supplementary Materials

The following supporting information can be downloaded at: https://www.mdpi.com/article/10.3390/insects15010013/s1, Figure S1: Chromatogram of volatiles (collected with HS-SPME) and extracts with n-hexane of *Menochilus sexmaculatus*; Table S1: Compounds of *Menochilus sexmaculatus* collected with HS-SPME; Table S2: Compounds of *Menochilus sexmaculatus* extracted with n-hexane; Table S3: Alkane compounds that made up 1% or more of the extracts with n-hexane and volatiles with HS-SPME of *Menochilus sexmaculatus*.
