# Supplementary material for: Predation Risk Effects of Lady Beetle Menochilus sexmaculatus (Fabricius) on the Melon Aphid, Aphis gossypii Glover
Source: Insects. 2023 Dec 27;15(1):13. doi: 10.3390/insects15010013 (PMC10816753; doi:10.3390/insects15010013)
Supplement: Supplementary file 1 [file insects-15-00013-s001.zip › insects-2725121-supplementary.pdf]

Supplementary Table S1. Compounds of *Menochilus sexmaculata* collected with HS-SPME

Number	Compound	CAS	Exist
1	Anethole	104-46-1	M、F、3
2	(+)-Longicyclene	1137-12-8	3
3	(-)-isolongifolene	1135-66-6	M、F
4	(+)-Longifolene	475-20-7	M、F、3
5	Caryophyllene	87-44-5	3
6	1,6-anhydro-β-D-glucose	498-07-7	3
7	Precocinelline	38211-56-2	M、F、3
8	2,6-Di-tert-butylpyridine	585-48-8	M、F
9	7-Isoquinolinol, 3,4-dihydro-6-methoxy-1-methyl-	4602-71-5	F
10	Adenine	73-24-5	3
11	N,N-dimethylhexadecylamine	112-69-6	3、F
12	2,5 – Dimethyl – 7 – hydroxy chromone	38412-47-4	3
13	n-Tricosane	638-67-5	M
14	n-Pentacosane	629-99-2	M、3
15	1-Heptacosanol	2004-39-9	M

Note: In the exist column, "M" indicates the males, "F" indicates the females t, and "3" indicates the third-instar larvae.

Supplementary Table S2. Compounds of *Menochilus sexmaculata* extracted with n-Hexane

Number	Compound	CAS	Exist
1	Ethylbenzene	100-41-4	3
2	m-Xylene	108-38-3	F、M、3
3	Isonicotinic acid, 2-phenylethyl ester	1000308-36-3	F、M、3
4	Hexanoic acid	142-62-1	3
5	1,4-cineole	470-67-7	3
6	2-Cyclohexen-1-ol,3-methyl-6-(1-methylethyl)-, 1-acetate	1204-30-4	3
7	2-Isopropyltoluene	527-84-4	3
8	(+)-Limonene	5989-27-5	3
9	Cineole	470-82-6	3

10	3,5-dichlorotoluene	25186-47-4	3
11	2-Ethylhexyl acrylate	103-11-7	M、3
**12**	**Precocinelline**	**38211-56-2**	**F、M、3**
**13**	**7-Isoquinolinol, 3,4-dihydro-6-methoxy-1-methyl-**	**4602-71-5**	**F、3**
**14**	**n-Pentacosane**	**629-99-2**	**F、M、3**
15	Ethyl Palmitate	628-97-7	3
16	n-Heneicosane	629-94-7	F、M
17	Oleic acid	112-80-1	F、M
18	Ethyl linoleate	544-35-4	M、3
19	Ethyl Oleate	111-62-6	M、3
20	n-Docosane	629-97-0	F、M
**21**	**n-Tricosane**	**638-67-5**	**F、M**
22	Z-12-Pentacosene	1000131-09-4	M
23	Glycidyl oleate	1000383-37-7	F、M
24	2-Methylpentacosane	629-87-8	3
25	Pentacosane, 3-methyl-	6902-54-1	F、M
**26**	**1-Heptacosanol**	**2004-39-9**	**F、M**
27	n-Hentriacontane	630-04-6	F、M
28	1-Hexacosanol	506-52-5	F、M
29	cis-13-Docosenol	629-98-1	F、M

Note: In the exist column, "M" indicates the males, "F" indicates the females, and "3" indicates the third-instar larvae. The bold components are also separated by solid phase microextraction.

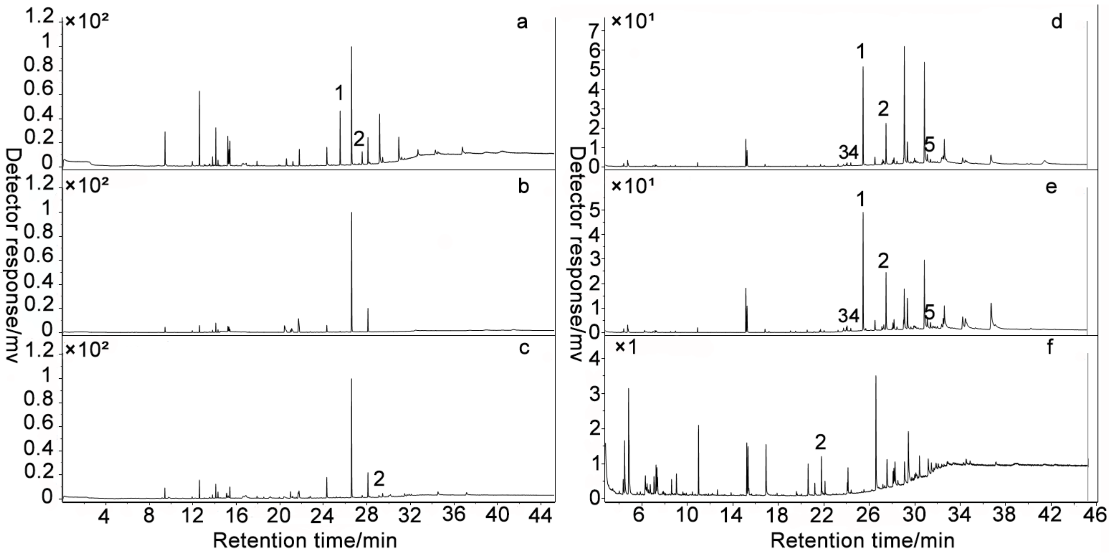

Supplementary Figure S1. Chromatogram of volatiles (collected with HS-SPME) and extracts with n-hexane of *Menochilus sexmaculatus.* Note: A, B and C are volatiles of male, female and third-instar larvae extracted by solid phase microextraction; D, E and F are male, female and

third-instar larvae extracted by n-hexane.

Supplementary Table S3. Alkane compounds that made up 1 % or more of the extracts with n-hexane and volatiles with HS-SPME of *Menochilus sexmaculatus*

Peak number[a]	Compound	Carbon number
1	n-Tricosane	23
2	n-Pentacosane	25
3	n-Henicosane	21
4	n-Docosane	22
5	n-Hentriacontane	31

Note: aPeak numbers correspond to labeled peaks in chromatogram of in Supplementary Figure S1.
